# A Novel Homozygous *VPS13B* Splice-Site Mutation Causing the Skipping of Exon 38 in a Chinese Family With Cohen Syndrome

**DOI:** 10.3389/fped.2021.651621

**Published:** 2021-04-20

**Authors:** Liangshan Li, Xiangmao Bu, Yuhua Ji, Ping Tan, Shiguo Liu

**Affiliations:** ^1^Medical Genetic Department, The Affiliated Hospital of Qingdao University, Qingdao, China; ^2^Department of Clinical Laboratory, Medical College of Qingdao University, Qingdao, China; ^3^Department of Transfusion, Qingdao Women and Children's Hospital, Qingdao, China; ^4^Newborn Disease Screening Centre, Yantai Maternal and Child Health Hospital, Yantai, China; ^5^Obstetrical Department, The Affiliated Hospital of Qingdao University, Qingdao, China

**Keywords:** cohen syndrome, VPS13B, splice-site mutation, mRNA analysis, exon skipping

## Abstract

**Background:** Cohen syndrome (CS) is a clinically heterogeneous disorder characterized by extensive phenotypic variation with autosomal recessive inheritance. *VPS13B* was identified to be the disease-causing gene for CS. The objectives of the present study were to screen likely pathogenic mutations of the patient with developmental delay and mental retardation, and to determinate the effect of this splice-site mutation by reverse transcription analysis.

**Methods:** Whole exome sequencing (WES) in combination with Sanger sequencing were performed to identify the causative mutations of this CS family. Subsequently, the impact of the intronic variant on splicing was analyzed by reverse transcription and the construction of expression vector.

**Results:** A novel homozygous splice-site mutation (c.6940+1G>T) in the VPS13B gene was identified in this proband. Sanger sequencing analysis of the cDNA demonstrated that the c.6940+1G>T variant could cause the skipping of entire exon 38, resulting in the loss of 208 nucleotides and further give rise to the generation of a premature in-frame stop codon at code 2,247.

**Conclusions:** The homozygous *VPS13B* splicing variant c.6940+1G>T was co-segregated with the CS phenotypes in this family and was identified to be the cause of CS after comprehensive consideration of the clinical manifestations, genetic analysis and cDNA sequencing result.

## Introduction

Cohen syndrome (CS) (OMIM 216550), initially described in three patients by Cohen et al., is an uncommon autosomal recessive neurodevelopmental disorder with more than 200 causative mutations in ~1,000 CS-affected individuals reported to date worldwide (1–3). CS can affect multiple organs and systems including the face, head, eyes, blood system, cardiovascular system, nervous system, and endocrine system (4). CS is relatively common among Finnish population in spite of the low prevalence worldwide (5). Apart from this, CS has also been reported in Indian, Jordanian, Chinese, Saudi, Tunisian, Iranian, German, Syrian, Lebanese, and Pakistani (6–14).

To date, clear phenotype–genotype correlations of CS have not been established yet. Although CS-affected individuals from outside Finland present with variable phenotypes (15), the typical clinical characteristics usually include intellectual disability, short stature, a cheerful disposition, retinal dystrophy, hypotonia, scoliosis, joint laxity, intermittent neutropenia, slender fingers, hyperlinear palms, midchildhood onset truncal obesity and craniofacial dysmorphisms such as microcephaly, thick hair, low hairline, short philtrum, wave-shaped eyes and prominent upper central incisors (1, 16, 17).

Vacuolar protein sorting 13 homolog B (*VPS13B*), also known as *COH1*, was identified to be the disease-causing gene for CS by Kolehmainen et al. (15) and since then, a large number of variants have been detected in CS patients. In addition, *VPS13B* is also responsible for autism spectrum disorders (ASDs) (18). *VPS13B* is localized on chromosome 8 (8q22.2) with 62 exons and encodes a 4022-amino acid transmembrane protein (14). The encoded protein is a Golgi-associated peripheral membrane protein that plays an important role in Golgi integrity and homeostasis, and membrane transport (17, 19), and it belongs to the VPS13 protein family, which are highly conserved in eukaryotic cells (20, 21). Loss-of-function mutations in other *VPS13* family members such as *VPS13A, VPS13C*, and *VPS13D* could result in chorea-acanthocytosis (OMIM 200150), rapidly progressive, early-onset autosomal recessive Parkinson's disease (OMIM 616840) and spinocerebellar ataxia, recessive type 4 (OMIM 607317), respectively (22–24).

With the rapid development of high-throughput sequencing technology, next-generation sequencing (NGS) for molecular analysis has enabled patients with inconspicuous clinical symptoms to get timely and accurate diagnosis, which could contribute to improving the quality of life of the patients and facilitating genetic counseling. NGS technology has been routinely available in clinical practice and research due to significant advantages including high efficiency, low cost, and high accuracy (25). Furthermore, it is also worth noting that NGS technology as a powerful tool is increasingly widely used in the identification of pathogenic mutations of rare monogenic disorders and the discovery of novel causative genes of certain diseases.

In the present study, we investigated a pedgree with CS from Shandong province, China and identified a novel homozygous splicing mutation in the VPS13B gene by performing trio-based whole-exome sequencing (WES). In addition, we further determined that the intronic mutation could lead to aberrant mRNA splicing by reverse transcription analysis.

## Materials and Methods

### Patient

**Figure 1:**
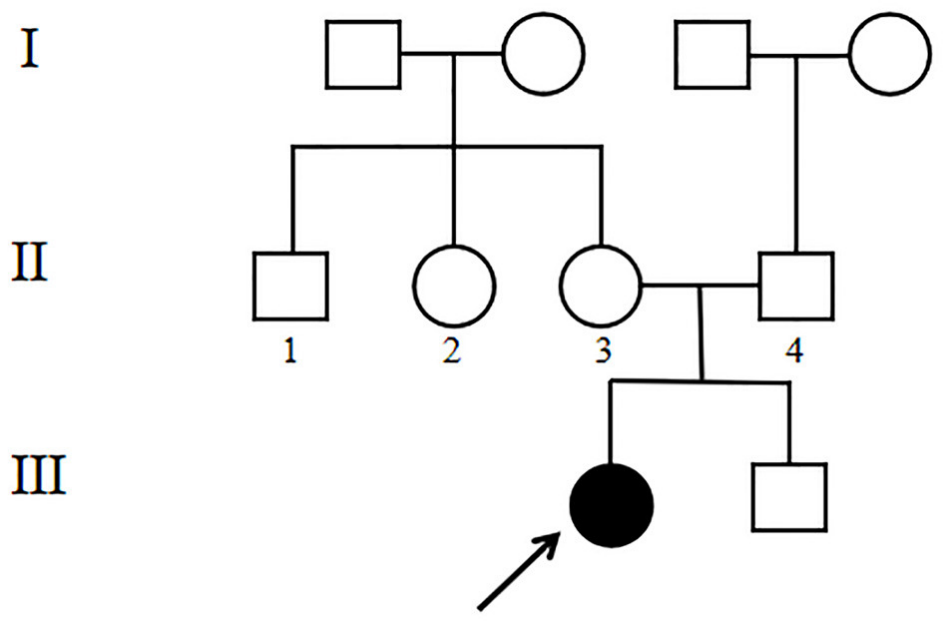
Pedigree of the family with CS. The black arrow denotes the proband.

The 4-year-old female proband (III1, Figure 1) was the first child of the family born at 40 weeks gestation with a birth weight of 2,600 g from non-consanguineous, healthy parents. There was no significant family history. Other family members including her younger brother (III2, Figure 1) did not show any obvious symptoms or signs. This study was approved by the Ethics Committee of the Affiliated Hospital of Qingdao University. Blood samples were collected from the proband and her family members after written informed consent was obtained from the parents.

### WES for Mutation Screening

The genomic DNA was isolated from peripheral blood leukocytes of the proband using a DNA extraction kit (TIANGEN, Beijing, China) following the manufacturer's protocol. The DNA was quantified with Nanodrop 2000. The qualified genomic DNA sample was randomly fragmented into 180–250 bp by Covaris S220 sonicator. DNA fragments were end repaired, A-tailed and ligated to adapters on both ends for the preparation of DNA libraries. Adapter-ligated libraries were enriched by polymerase chain reaction (PCR) amplification. A Agilent 64 M liquid phase chip capture system was used to efficiently enrich the whole-exome regions. Exome libraries were enriched in a PCR reaction followed by library quality assessment. Only qualified libraries were sequenced on Illumina NovaSeq platform for paired-end 150 bp reads. The target area coverage was 99.73%, the average sequencing depth was 189.58 × and the proportion of average depth of target area >20 × was 98.97%.

### Sequencing Data Analysis

For raw data, filter reads with adapter contamination, reads with more than 10% of uncertain bases and low quality reads to obtain clean data. Burrows-Wheeler Aligner (BWA) software was used to compare clean reads with reference genome (GRCh37/hg19). Samtools and picard tool were utilized to sort the comparison results and mark duplicate reads, respectively. Single nucleotide polymorphisms (SNPs) and insertions or deletions (InDels) were determined by the Genome Analysis Toolkit (GATK) software. Subsequently, the SNPs and InDels were annotated by ANNOVAR. Remove synonymous variants and variants with minor allele frequency (MAF) > 1% in at least one of the three available frequency databases 1000 Genomes Project, Exome Aggregation Consortium (ExAC) and esp6500si_all. Finally, the pathogenicity of the variants were predicted by SIFT (https://sift.bii.a-star.edu.sg/), PolyPhen-2 (http://genetics.bwh.harvard.edu/pph2/), MutationTaster (http://www.mutationtaster.org/) and CADD (https://cadd.gs.washington.edu/score).

### Sanger Sequencing Validation

The identified variant by WES analysis was confirmed by Sanger sequencing. The genomic DNA was extracted from peripheral blood samples of the proband, her parents, paternal grandparents and maternal grandparents with a DNA extraction kit (TIANGEN, Beijing, China). The partial DNA sequences involving the splicing mutation site were amplified by PCR using primers: forward (5′-TTAATGAGGAGGGAAATTTTGAAGTAC-3′) and reverse (5′-TGGGCAATCTTCAGTTTCATTATAAA-3′). PCR products were analyzed by 1% agarose gel electrophoresis and then were purified and sequenced on an ABI 3730 analyzer (Applied Biosystem). The obtained DNA sequences were compared with the reference sequence on National Center Biotechnology Information (NCBI) website to discover the mutation site.

### mRNA Analysis by the Construction of Recombinant Plasmid

Total RNA was extracted from peripheral venous blood of the proband using a Blood RNA Extraction Kit (Takara) according to the manufacturer's instructions. cDNA was prepared from 2 μg total RNA using HiScript® II 1st Strand cDNA Synthesis Kit (+gDNA wiper) (Vazyme). PCR for amplification of the cDNA covering exons 37–41 and partial sequences of exons 36 and 42 of the VPS13B gene was performed with 2× TransStart® FastPfu PCR SuperMix (TransGen Biotech) following primers: forward (5′-CAAGAAAACATGTGGAGAGCTGTT-3′) and reverse (5′-CACTGTCGAAGATACATGTGTGGTT-3′). PCR product was identified by 1% agarose gel electrophoresis followed by the extraction of target cDNA with an agarose gel DNA recovery kit (Solarbio). Subsequently, the recovered PCR product was connected with pEASY®-Blunt E2 Expression Vector. The recombinant plasmid was confirmed by bidirectional sequencing with universal primers: forward (5′-TAATACGACTCACTATAGGG-3′) and reverse (5′-TAGTTATTGCTCAGCGGTGG-3′).

## Results

### Clinical Phenotypes

On October 30, 2017, when the proband was 1 year and 3 months old, she was admitted to hospital because she was unable to stand or walk independently. Physical examination showed good eye contact, decreased muscle tone in all limbs, active patellar tendon reflex, ankle clonus (–), hyperextended knee and talipes valgus (Table 1). Preliminary diagnoses were growth retardation and hypotonia. She could speak “ba” and “ma” at the age of 2 years and was able to walk with an unsteady gait at age 3 years.

**Table 1:** Summary of clinical findings of the proband.

**Clinical phenotypes**	**Patient (III1)**
Developmental delay	+
Intellectual disability	+
Hypotonia	+
Micrognathia	+
Wave-shaped eyes	+
Short philtrum	+
Thick hair	+
Thick eyelashes	+
High myopia	+
Neutropenia	+
Talipes valgus	+
Left coherent palm	+
Hyperlinear palms	+

**Figure 2:**
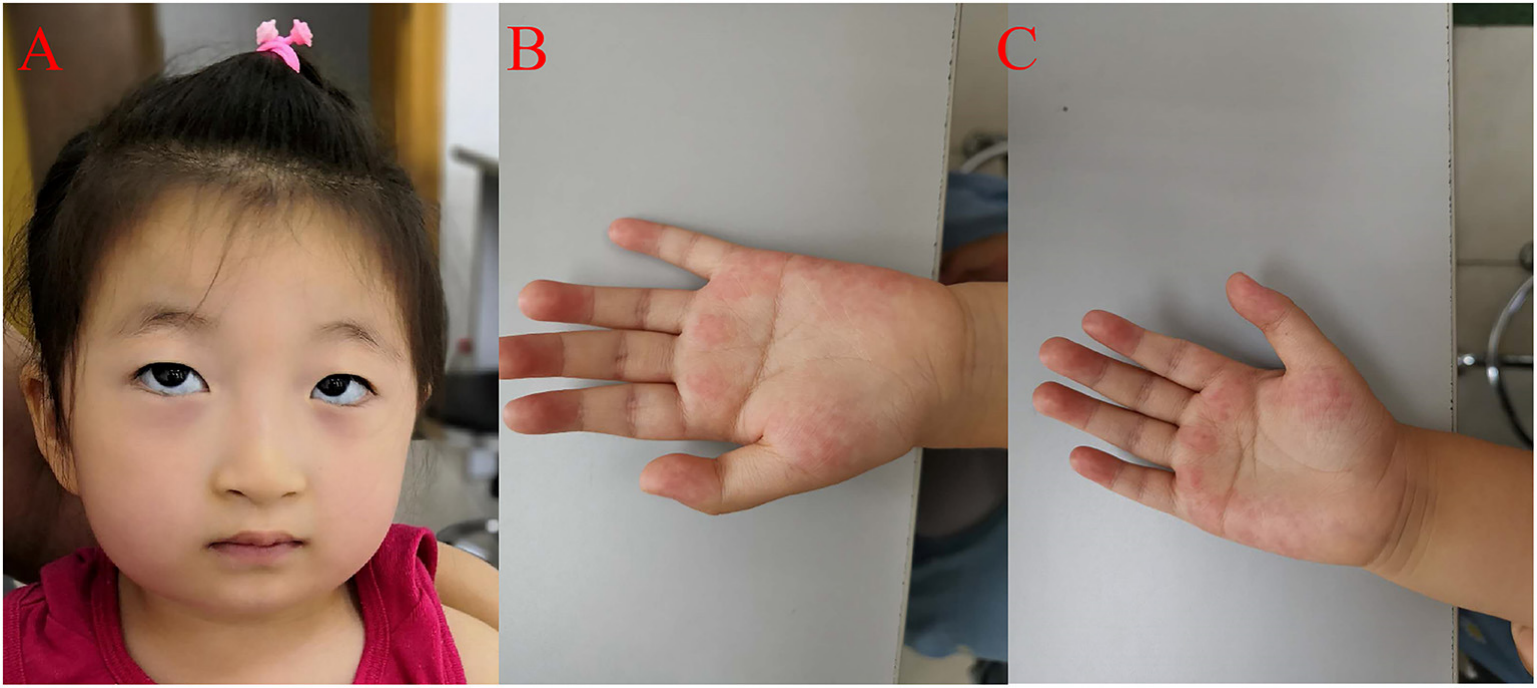
Clinical features of the proband. **(A)** The proband presented with micrognathia, wave-shaped eyes, short philtrum, thick eyelashes and thick hair. **(B)** Left coherent palm with hyperlinearity. **(C)** Hyperlinear right palm.

She was readmitted to hospital due to growth and development retardation, and short stature on May 20, 2020 when she was 3.8 years old. Physical examination: weight 13.4 kg, length 94.7 cm (3rd−10th centile), normal stature, micrognathia, normal limbs and spine, left coherent palm, bipedal varus, unlimited joint movements, no edema in both lower limbs, decreased muscle strength and muscle tension of the limbs, normal bilateral patellar tendon reflexs and bilateral Babinski signs (–) (Figure 2; Table 1). Laboratory tests: neutrophil count 1.16 × 10^9^/L (Reference value: 1.7–7.7 × 10^9^/L), urine occult blood (±), urine leukocyte (±), cortisol 213.35 nmol/L (Reference value: 118.6–618 nmol/L), adrenocorticotropic hormone (ACTH) 18.00 pg/mL (Reference value: 0–46 pg/mL), free triiodothyronine (FT3) 5.36 pmol/L (Reference value: 3.5–6.5 pmol/L uIU/mL), free thyroxine (FT4) 13.92 pmol/L (Reference value: 11.5–22.7 pmol/L), thyroid stimulating hormone (TSH) 1.722 μIU/mL (Reference value: 0.64–6.27 uIU/mL), insulin-like growth factor 1 (IGF1) 116 μg/L (Reference value: 49–289 μg/L), 25-hydroxyvitamin D 14.31 ng/mL (Reference value: 20–100 μg/L), estradiol 26.66 pmol/L (Reference value: 22–99.1 pmol/L), luteinizing hormone <0.10 mIU/mL (Reference value: 0.2–1.4 mIU/mL), follicle-stimulating hormone 0.52 mIU/mL (Reference value: 0.2–3.8 mIU/mL). The measured growth hormone (GH) values were 0.16, 6.9, 3.0, and 0.16ng/mL, 2.4, 1.9, respectively, at 0, 60, and 90 min on the GH provocation test by oral clonidine and insulin injection with GH peak <10 ng/mL. Blood glucose levels were 5.6 mmol/L, 5.4 mmol/L, 5.5 mmol/L at 0, 60, and 90 min. Plain magnetic resonance imaging (MRI) scan of the pituitary showed no obvious abnormality. Orthotopic radiograph of the left hand revealed that the bone age was equivalent to about 3.5 years of age. No abnormalities were found in hepatobiliary, pancreatic and splenic ultrasound. Abdominal gynecologic ultrasound revealed uterus 15^*^4 cm, left ovary 11^*^5 cm, and right ovary 14^*^4cm.

The patient had a normal 46, XX chromosome karyotype and a normal arr (1–22, X)×2 chromosomal microarray. She suffered from intermittent neutropenia with data in different periods shown in Table 2. Furthermore, she exhibited mental retardation, wave-shaped eyes, short philtrum, thick hair, thick eyelashes, high myopia (OD −2.10DS −1.19DC^*^84, OS −3.18DS −2.90DC^*^74), hyperlinear palms (Figure 2; Table 1). Genetic testing on this proband was performed for further diagnosis.

**Table 2:** Neutrophil counts in different periods.

	**2017-11-8**	**2017-12-17**	**2019-1-19**	**2019-7-3**	**2020-5-20**	**2020-5-21**	**2020-6-18**
Neutrophil count (Reference value: 1.7–7.7 × 10^9^/L)	1.36 × 10^9^/L	1.01 × 10^9^/L	1.91 × 10^9^/L	1.9 × 10^9^/L	1.16 × 10^9^/L	0.87 × 10^9^/L	1.52 × 10^9^/L

### Genetic Analysis

**Figure 3:**
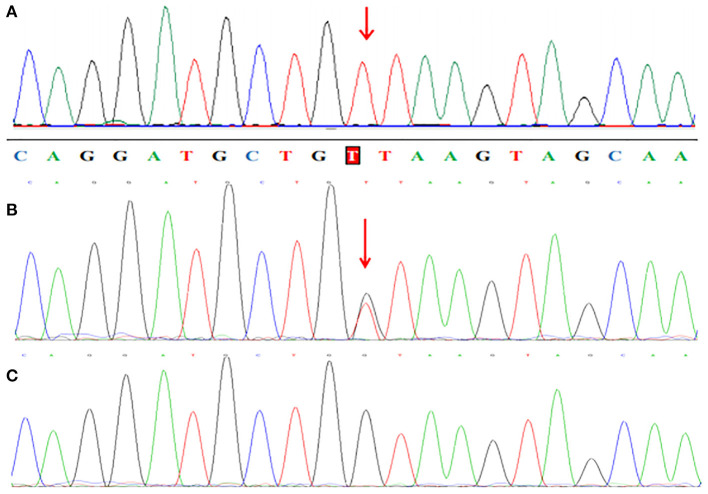
Partial sequence chromatograms of *VPS13B*. The red arrows represent the mutation site. **(A)** Homozygous c.6940+1G>T splice-site mutation. **(B)** Heterozygous c.6940+1G>T splice-site mutation. **(C)** Normal DNA sequence.

WES analysis revealed a novel homozygous splice-site *VPS13B* mutation (c.6940+1G>T, rs202046738) (NM_017890.5) in this proband (III1, Figures 1, 3A), which resulted in the first base of intron 38 changed from G to T. Several bioinformatics analysis tools were used to predict the deleteriousness of this splice donor site mutation. MaxEntScan (http://hollywood.mit.edu/burgelab/maxent/Xmaxentscan_scoreseq.html) showed Maximum Entropy Model (MAXENT) 2.14, Maximum Dependence Decomposition Model (MDD) 7.37, First-order Markov Model (MM) 1.73, Weight Matrix Model (WMM) 2.53, with scores of 10.65, 15.88, 10.24, and 11.04, respectively, in wild type. Spliceman (http://fairbrother.biomed.brown.edu/spliceman/index.cgi) revealed a ranking of 59% and Alternative Splice Site Predictor (ASSP) (http://wangcomputing.com/assp/index.html) revealed a score of 5.600 (Donor site cutoff: 4.5). The pathogenicity of the splicing variant was classified as “likely pathogenic” (PVS+PM) according to the American College of Medical Genetics and Genomics (ACMG) guidelines (26) and the frequencies of this mutation were 0.019968% in 1,000 g2015aug_all, 0.01% in gnomAD_exome_EAS, 0.0008% in gnomAD_exome_ALL. Molecular analysis indicated that the homozygous variant was inherited from the unaffected parents (Figure 3B). Her maternal grandfather (I1, Figure 1) and paternal grandfather (I3, Figure 1) were heterozygotes for this variant (Figure 3B). However, the maternal grandmother (I2, Figure 1) and paternal grandmother (I4, Figure 1) did not carry the mutation (Figure 3C). In addition, the mutation site was not found in 100 unrelated healthy controls in Shandong, China, either (Figure 3C). Therefore, the homozygous genotype was co-segregated with the CS phenotypes in this family.

### mRNA Analysis for the VPS13B Splicing Variant

**Figure 4:**
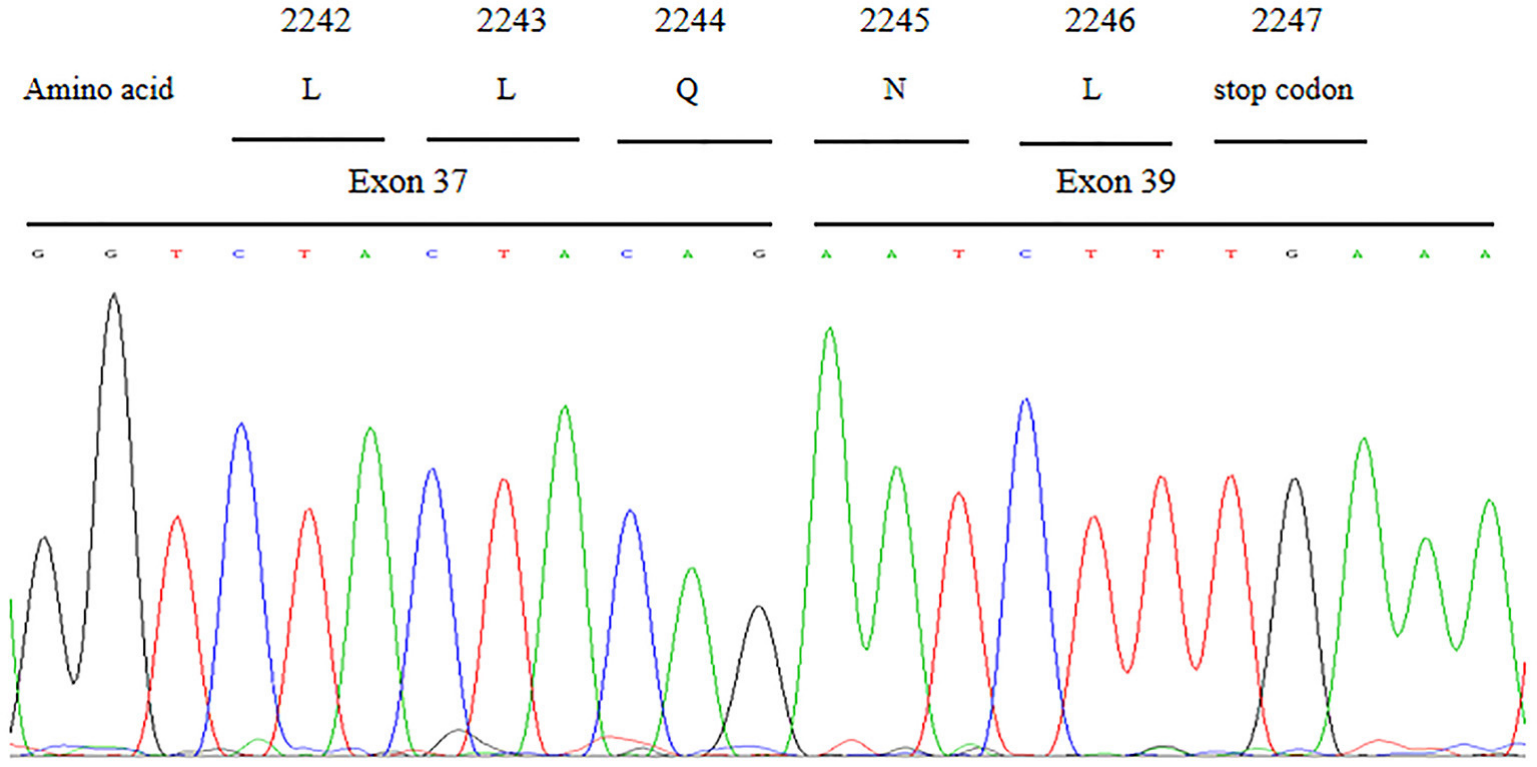
Partial cDNA sequence chromatograms of *VPS13B*. The aberrant splicing resulted in the skipping of entire exon 38 and abnormal direct joining of exon 37 and exon 39, which further gave rise to the generation of a premature in-frame stop codon at code 2,247.

cDNA sequence analysis confirmed that the c.6940+1G>T variant could result in aberrant splicing which caused the skipping of entire exon 38 and abnormal direct joining of exon 37 and exon 39 (Figure 4). The skipping of exon 38 led to loss of 208 nucleotides and further gave rise to the generation of a premature in-frame stop codon at code 2247. Presumably, the c.6940+1G>T variant in *VPS13B* is responsible for functional defect of the truncated protein.

## Discussion

In this study, we identified a novel homozygous splicing *VPS13B* variant c.6940+1G>T in this proband by high-throughput sequencing analysis, which was inherited from both parents who were non-consanguineous. Furthermore, the mutation frequency was extremely low in different databases, we therefore speculated that the homozygous *VPS13B* variant in this proband may be explained by the founder effect. Although this mutation locus in heterozygous status has been described (27), we reported here a homozygous c.6940+1G>T mutation for the first time to our knowledge. In 2020, Lou et al. also described two CS sisters with heterozygous c.6940+1G>T variant and demonstrated that the splice donor site mutation could result in the entire skipping of exon 38, the clinical features slightly differed from those of our patient (2). The proband in our study also presented with coherent palm with hyperlinearity, which has only been reported in Chinese population to date. The c.6940+1G>T variant was evaluated as “likely pathogenic” according to the ACMG guideline classification. Genetic analysis showed that her maternal grandfather and paternal grandfather were heterozygous carriers for this mutation, while it was absent in the maternal grandmother and paternal grandmother as well as 100 unrelated, healthy individuals of Chinese origin. Co-segregation analysis suggested that this splice-site mutation was likely responsible for the CS phenotypes in this family. To determine the effect of this *VPS13B* splicing variant, total RNA isolated from venous blood sample was reverse transcribed for the construction of expression vector. Sanger sequencing analysis of the cDNA covering exons 36–42 of *VPS13B* showed that the homozygous c.6940+1G>T variant could cause the skipping of entire exon 38, which resulted in a premature stop codon producing a truncated VPS13B protein. The homozygous c.6940+1G>T variant in *VPS13B* was identified to be the cause of CS after comprehensive consideration of the clinical manifestations, genetic analysis and cDNA sequencing result.

As a rare autosomal recessive developmental disorder with a broad phenotypic spectrum, CS has been reported in different populations (28). It is characterized by stunted growth, intellectual disability, short philtrum, hypotonia, truncal obesity, overly sociable behavior, early onset and severe myopia, microcephaly, intermittent neutropenia (neutrophil count <1.5 × 10^9^/L in children and <1.8 × 10^9^/L in adults) (29, 30). The clinical features of the proband in our study were consistent with typical characteristics of CS. The early clinical diagnosis of CS remains challenging and the diagnostic criteria are controversial due to the overlapping features with other disorders and the clinical heterogeneity of CS. The incidence rate of CS may be higher than 1:105,000 because certain clinical symptoms are insignificant during early childhood, which results in CS patients not being diagnosed in a timely manner (31).

CS has been attributed to loss-of-function biallelic mutations in the VPS13B gene. Since the founder mutation c.3348_3349delCT was found in Finnish patients, more than 200 causative mutations have been reported so far in ~1,000 CS-affected individuals worldwide including non-sense, duplication, missense, splicing, insertion/deletion mutations (7, 15, 17). *VPS13B* maps to chromosome 8q22.2 and encodes six protein isoforms generated by alternative splicing (https://www.uniprot.org/). VPS13B is a transmembrane protein that is associated with vesicle-Mediated sorting, intracellular protein transport, Golgi glycosylation and morphology, and lysosomal–endosomal pathway maintenance (15, 32). VPS13B is widely expressed in brain, blood, small intestine, muscles, placenta, heart, retina, kidney and lung (13, 30).

Rejeb et al. described two cases affected by CS from a non-consanguineous family for the first time in the Tunisian population in 2017 with the clinical features of neutropenia, mental retardation, tapering fingers, thick hair eyebrows and lashes (13). Novel compound heterozygous *VPS13B* mutations c.3582delT/p.A1149fs and c.6295_6296delAT/p.M2124fs were found in the two siblings, which were inherited from the father and the mother, respectively. In 2007, Katzaki et al. identified a deletion variant c.11125delC/p.T3708 fsX61 and a non-sense variant c.11314C>T/p.Q3772X in a male Italian patient with CS who presented with truncal obesity with BMI of 32.2, severe intellectual disability, typical facial gestalt, retinopathy, myopia, joints hyper extensibility, neutropenia and tapering fingers (33). In 2006, six CS cases carrying the same homozygous c.4471G>T/p.Glu1491X in *VPS13B* were reported by Murphy and her colleagues. The patients manifested microcephaly, short philtrum, truncal obesity, developmental delay and prominent central incisors, which were consistent with typical phenotypes of CS (34).

CS is less frequent among Chinese population with only several cases reported to date. In 2019, two CS siblings from Chinese healthy, non-consanguineous parents exhibited mental retardation, speech delay, microcephaly, generalized joint hyper extensibility, hypotonia, thick hair, thick eyebrows, prominent upper central incisors, and hyperlinear palms (14). Hyperlinear palms is an additional phenotypic characteristic of CS only described in Chinese population and the clinical features of our patient have confirmed this. In addition, novel splicing maternal mutation c.3666+1G>T and novel non-sense paternal mutation c.9844A>T/p.K3282X in *VPS13B* were identified in the two siblings by performing WES.

In summary, we identified a novel homozygous splice-site mutation c.6940+1G>T in *VPS13B* by performing WES in a proband with CS. The effect of this splicing variant was confirmed by Sanger sequencing of the cDNA combined with *in silico* analysis that the aberrant splicing led to the skipping of entire exon 38. Family study has revealed that the *VPS13B* variant was co-segregated with the CS phenotypes in this family. Our research demonstrated the pathogenicity of this c.6940+1G>T mutation and made great contributions to the establishment of the genotype–phenotype correlations of CS.

## Data Availability Statement

The data presented in the study are deposited in the ClinVar repository, accession number SCV001499947.

## Ethics Statement

This study was approved by the Ethics Committee of the Affiliated Hospital of Qingdao University (QDFY20208902). Written informed consent to participate in this study was provided by the participants' legal guardian/next of kin.

## Author Contributions

LL conducted the experiments and drafted the manuscript. XB analyzed the data. YJ analyzed and critically reviewed the manuscript. PT drafted the design. SL revised the manuscript. All authors read and approved the final manuscript.

## Conflict of Interest

The authors declare that the research was conducted in the absence of any commercial or financial relationships that could be construed as a potential conflict of interest.

## Abbreviations

ACMG: American College of Medical Genetics and Genomics
ACTH: Adrenocorticotropic hormone
ASDs: Autism spectrum disorders
CS: Cohen syndrome
FT3: Free triiodothyronine
FT4: Free thyroxine
GH: Growth hormone
IGF1: Insulin-like growth factor 1
MRI: Magnetic resonance imaging
NCBI: National Center Biotechnology Information
NGS: Next-generation sequencing
PCR: Polymerase chain reaction
TSH: Thyroid stimulating hormone
*VPS13B*: Vacuolar protein sorting 13 homolog B
WES: Whole exome sequencing.
